# An ultra-high-density map as a community resource for discerning the genetic basis of quantitative traits in maize

**DOI:** 10.1186/s12864-015-2242-5

**Published:** 2015-12-21

**Authors:** Hongjun Liu, Yongchao Niu, Pedro J. Gonzalez-Portilla, Huangkai Zhou, Liya Wang, Tao Zuo, Cheng Qin, Shuaishuai Tai, Constantin Jansen, Yaou Shen, Haijian Lin, Michael Lee, Doreen Ware, Zhiming Zhang, Thomas Lübberstedt, Guangtang Pan

**Affiliations:** Maize Research Institute of Sichuan Agricultural University/Key Laboratory of Biology and Genetic Improvement of Maize in Southwest Region, Ministry of Agriculture, 611130 Chengdu, China; BGI-Shenzhen, Shenzhen, 518083 China; Department of Agronomy, Iowa State University, Ames, IA 50011 USA; Cold Spring Harbor Laboratory and USDA: USDA ARS NAA Robert W. Holley Center for Agriculture and Health, Agricultural Research Service, 14853 Ithaca, NY USA; Interdepartmental Genetics Graduate Program, Iowa State University, Ames, 50011 USA

**Keywords:** IBM Syn10, Resequencing, iPlant Discovery Environment, Quantitative trait locus mapping, Inadvertent selection

## Abstract

**Background:**

To safeguard the food supply for the growing human population, it is important to understand and exploit the genetic basis of quantitative traits. Next-generation sequencing technology performs advantageously and effectively in genetic mapping and genome analysis of diverse genetic resources. Hence, we combined re-sequencing technology and a bin map strategy to construct an ultra-high-density bin map with thousands of bin markers to precisely map a quantitative trait locus.

**Results:**

In this study, we generated a linkage map containing 1,151,856 high quality SNPs between Mo17 and B73, which were verified in the maize intermated B73 × Mo17 (IBM) Syn10 population. This resource is an excellent complement to existing maize genetic maps available in an online database  (iPlant, http://data.maizecode.org/maize/qtl/syn10/). Moreover, in this population combined with the IBM Syn4 RIL population, we detected 135 QTLs for flowering time and plant height traits across the two populations. Eighteen known functional genes and twenty-five candidate genes for flowering time and plant height trait were fine-mapped into a 2.21–4.96 Mb interval. Map expansion and segregation distortion were also analyzed, and evidence for inadvertent selection of early flowering time in the process of mapping population development was observed. Furthermore, an updated integrated map with 1,151,856 high-quality SNPs, 2,916 traditional markers and 6,618 bin markers was constructed. The data were deposited into the iPlant Discovery Environment (DE), which provides a fundamental resource of genetic data for the maize genetic research community.

**Conclusions:**

Our findings provide basic essential genetic data for the maize genetic research community. An updated IBM Syn10 population and a reliable, verified high-quality SNP set between Mo17 and B73 will aid in future molecular breeding efforts.

**Electronic supplementary material:**

The online version of this article (doi:10.1186/s12864-015-2242-5) contains supplementary material, which is available to authorized users.

## Background

Maize is an important crop species and is widely used for food, feed, and renewable fuel production. Recently, there has been an increasing disparity between human population growth and crop yields [1]. Understanding the genetic control of trait architecture in maize is important and can accelerate the process of developing new varieties for the breeding community. In a modern breeding platform, a QTL mapping strategy is employed to efficiently identify chromosomal regions (genes/QTLs) contributing to agronomic traits [2–14]. Furthermore, with the advances of the B73 reference genome and a dramatic decrease in sequencing costs, the utilization of next-generation sequencing (NGS) for cost-efficient high-throughput genotyping has shown greater advantages compared to the use of traditional markers [15, 16]. However, as researchers focus on either basic research or different types of traits in maize breeding, developing a large mapping population with high recombinant rates is needed to detect even the small effect quantitative trait loci (QTLs) and positional cloning of underlying genes (http://www.maizegdb.org/cgi-bin/displaymaplistresults.cgi?term=%25). Different types of genetic maps can discern diverse agronomic traits in breeding programs; however, the genotype data are limited for overall comprehensive analysis.

In maize, B73 and Mo17 are the most widely studied inbred lines in two important opposing heterotic groups and are widely used in genetic studies throughout the world. Based on crossing and four generations of intermating, the intermated B73 × Mo17 RIL population (IBM Syn4) has served as a genetic reference mapping population for detecting QTLs [17–21] and integrating genetic and physical maps [22, 23]. Furthermore, Hussain et al. have constructed an update ten-generation intermated B73 × Mo17 doubled haploid population (IBM Syn10 DH population, Additional file 1), which exhibits a higher genetic resolution than the earlier Syn4 population version [24, 25], with an almost two-fold increase in the genetic map length. The phenotypic variation present within the IBM population has been used in QTL mapping studies and has served as the reference map in meta-analysis studies and thus is an important resource for the maize genetic research community [13, 17, 21, 26–29].

Currently, high-throughput re-sequencing strategies are being used to study accurate mapping QTLs, the history of maize domestication, and genome structural variations in modern breeding programs. Using re-sequencing technologies, Huang et al. [30] have developed an ultra-high-density linkage map by using a whole-genome re-sequencing and “bin marker” strategy. They aligned the SNPs of individual lines and grouped adjacent 100-kb intervals with the same genotype across the entire RIL population into a single recombination bin. Such recombination bins between two adjacent recombination breakpoints were defined as single “bin markers”. These markers have advanced the detection and calculation of true recombination breakpoints with thousands of bin markers, which can benefit QTL genome-wide analysis. The bin-mapping strategy has been shown to be superior in detecting and fine mapping QTLs versus traditional methods [31–33]. Using the same methods, Huang *et al.* [34] have constructed an updated bin map with 1,793 bin markers among 271 lines for QTL mapping. The identification of 58 QTLs, including ten known causal genes for selective sweeps, has improved understanding of rice domestication. In addition, Zhou *et al.* [35] have constructed an ultra-high-density SNP bin map by sequencing an “immortalized F_2_” population and calculated single-locus and epistatic genetic effects to further understand the biological mechanisms of heterotic performance in rice hybrids. Furthermore, by using a bin marker strategy, yield-associated loci in rice [36] and root-knot nematode resistance QTLs in soybean have been fine mapped [37], respectively.

In this study, we genotyped the IBM Syn10 population using bin map strategies and constructed a high-density map by taking advantage of their genetic diversity and bioinformatics analysis. This re-sequencing of the IBM Syn10 DH population will provide new insights into: 1) the high-quality SNP data by deeply re-sequencing the parental line Mo17 and analyzing the genome variation between B73 and Mo17, 2) an ultra-high-density SNP genotype dataset with thousands of recombination bins in the IBM Syn10 population, and 3) the construction of an integrated map with high-quality SNP, SSR, RFLP, IDP, and bin markers. Our results provide a broad-interest dataset for advanced forward genetics research and community resources, thus accelerate the molecular breeding process. Furthermore, there are huge computational burdens for the analysis of the various phenotype datasets, such as genetic linkage map construction and QTL mapping, due to a lack of suitable software for sequencing data. Thus, we parallelized the Composite Interval Mapping (CIM) function of QTL Cartographer, and integrated it into the iPlant Discovery Environment (DE) to reduce the bottleneck of large-scale QTL analysis [38]. Additionally, via the iPlant platform, we provide a readily accessible community resource to molecular breeding community.

## Results

### Mo17 re-sequencing and genome variation in comparison with B73

**Fig. 1:**
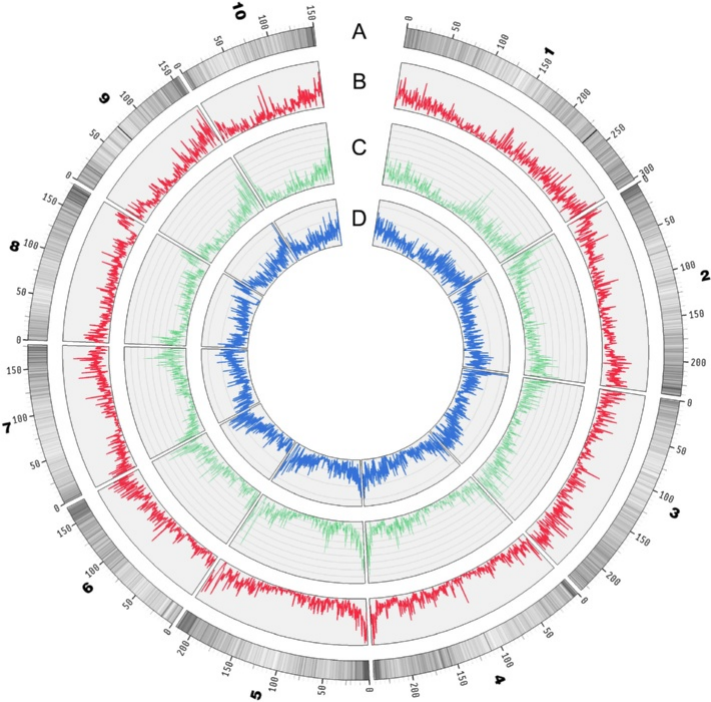
Genome-wide SNP distribution and genetic variation throughout the maize genome. **a**. Gene density of chromosomes is visualized by line darkness and the gene density is proportional to color depth. **b**. Density of genic SNPs on chromosomes are visualized by line redness. **c**. Density of SNPs in CDS (Coding Sequence) is visualized by line greenness. **d**. Density of Indels (insertions or deletions) are visualized by line blueness

In this study, we performed a deep re-sequencing of the inbred line Mo17 (~26.65×) to construct a high-quality set of SNPs with the B73 reference genome. Mo17 re-sequencing yielded 54.89 Gb of data. In total, 90.76 % of the clean reads were aligned to the B73 reference with 90.59 % coverage of the genome. In total, there were 3,097,838 SNPs between Mo17 and B73, and 2,200,187 SNP markers were homozygous with both parents but polymorphic between parents (Additional file 2). Of these homozygous SNP markers, 1,505,749 had uniquely physical coordinates in the B73 genome, and were retained for bin map construction, whereas the multiple-alignment SNPs that were inconsistent with the reference genome were discarded. The distribution of homozygous SNPs along the chromosomes indicated a high and uniform coverage across the whole genome (Additional file 3, 4 and 5). We also identified 180,587 indels that ranged from 1 bp to 5 bp in Mo17 relative to B73 reference, of which 82,996 (45.96 %) were insertions and 97,591 (54.04 %) were deletions; homozygous and heterozygous indel variations were 137,330 (76.05 %) and 43,257 (23.95 %), respectively (Additional file 6). The SNP and indel distribution are illustrated in Fig. 1.

### Genotype calling in IBM Syn10 and bin map construction

**Fig. 2:**
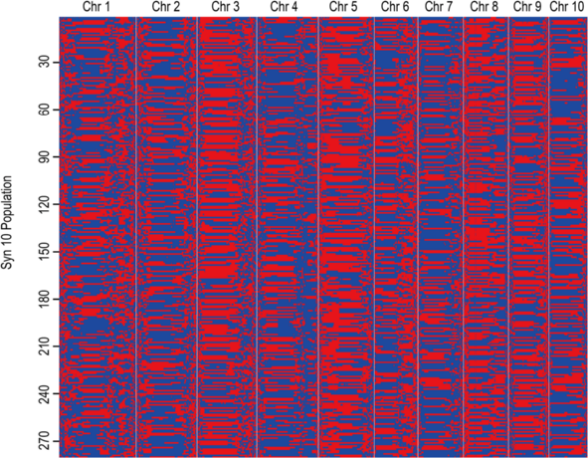
Recombination bin map of the IBM Syn10 population. Bin map consisting of 6,618 bin was inferred from 1,505,749 homologous high quality SNPs markers in the IBM Syn10 population. Physical positions are based on B73_RefGen_v2. Red, Mo17 genotype; Blue, B73 genotype

An average of ~ 640 Mb of data was generated for each IBM Syn10 line (ranging from 229.98 Mb to 1,252.97 Mb), which was equivalent to ~ 0.31× coverage of the maize genome. The detected SNPs were presented in Additional file 7. A bin map was constructed with 35,128 recombination breakpoints for all lines, in which each line contained an average of 125 breakpoints. The 280 individuals were merged into bin maps (Fig. 2) comprising 6,618 recombination bins, which captured the vast majority of the recombination events [30] (Additional file 8). The physical length per recombination bin ranged from 50 kb to 18.8 Mb with an average of 0.31 Mb. In total, 6,332 bins (95.68 %) were less than 1 Mb, 276 bins ranged from 1 Mb to 10 Mb in length, and only ten bins were in the range of 10–20 Mb. The detected bin numbers in our data were approximately a 4.09-fold (1,619 bins) [35] to 2.84-fold (2,334 bins) [30] increase compared with previous research on rice.

### Genetic linkage maps of the IBM Syn10 DH population

A high-density genetic map was constructed for the IBM Syn10 population with all 6,618 recombination bin markers [39]. The map quality was reliable as only adjacent markers showed strong linkage with 11,198.5 cM genetic distance in the map (Additional file 9). The average genetic distance of the bin markers was 1.7 cM, which represented an increase in the marker density compared with the 4.7 cM observed between the adjacent markers, for a total of 6,242.7 cM with 1,340 markers in the Syn4 population.

**Fig. 3:**
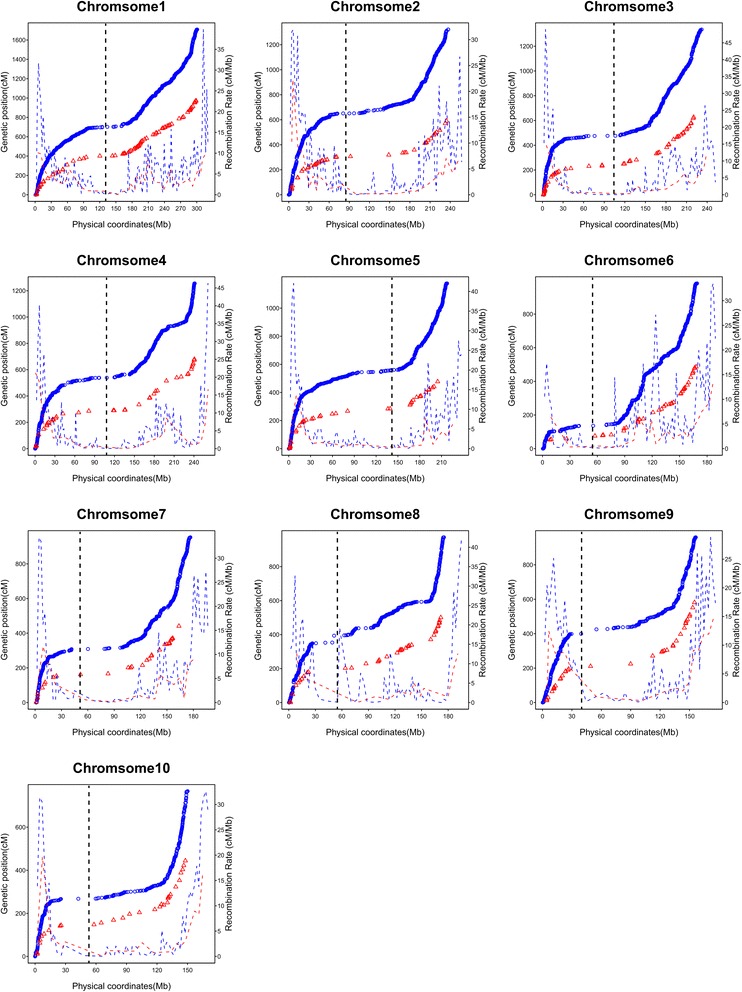
Plots of genetic *vs.* physical distance and corresponding recombination rates between the IBM Syn4 and Syn10 population. X-axis: physical position (in Mb) of the Bin/ SSR markers on the B73 physical map. Left Y-axis: genetic positions of Bin/SSR markers on IBM Syn10 (blue circles) and IBM Syn4 (red triangles) linkage maps. Right Y-axis: recombination rate in centiMorgen per Mega base pair (cM/Mb) for IBM Syn10 (dashed blue line) and IBM Syn4 (dashed red line). Recombination rate (cM/Mb) of each region was obtained as the quotient calculated from genetic and physical distance of two adjacent markers. Dashed black line in each chromosome represent the centromere physical position

Common features, such as the recombination rate and genetic *vs.* physical distance were compared between the Syn4 and Syn10 populations. Figure 3 shows the relationship between the genetic and physical positions on all chromosomes between the two populations. The recombination suppression was observed near the centromeres, and higher recombination rates were found to be predominant near both telomeres. The average ratio of genetic-to-physical distance was 6.95 cM/Mb for the whole genome, which is higher than that of the Syn4 population (4.55 cM/Mb).

The Syn10 genetic map could efficiently discover mis-assembled B73 genome. Ganal *et al.*[40] have used SNP arrays with 20,788 markers, which were genetically aligned into the IBM Syn4 map and physically placed on the B73 reference genome. These results have shown that several linked SNPs of chromosome 2 are erroneously positioned on chromosome 8. In our study, a 600-kb fragment composed of 4 bins was detected in IBM Syn10, and re-assigned from chromosome 8 to chromosome 2 due to an excess of double-crossover events. In consequence, bin markers chr08.482.5, chr08.484.5, chr08.487.5, and chr08.486.5 were not placed on chromosome 8 but instead were placed on chromosome 2 between 115 Mb and 118.5 Mb (Additional file 10), which reduced the number of double-crossover events. This finding, which was consistent with those in a previous report, indicated that the results are robust for *de novo* genetic mapping and reference map updating, although the bin markers were obtained from alignment-based procedures.

### Segregation distortion and map expansion

A total of 3,597 (54.4 %) markers in Syn10 showed distorted segregation (chi-squared test, *P* = 0.01) in contrast to 476 (35.5 %) markers in Syn4. Of the segregation distortion markers, 2,474 (37.4 %) of the markers in Syn10 showed a preference toward the genotype of B73 in contrast to 266 (19.9 %) in Syn4; the other 1,123(17.0 %) markers in Syn10 showed a preference toward Mo17, whereas only 210 (15.7 %) markers were observed in Syn4. Using the genetic composition survey, 176 (62.9 %) lines of Syn10 were found to be biased toward the genetic background of B73 in contrast to 94 (38.5 %) in Syn4, and 38 (13.6 %) lines of Syn10 were biased toward Mo17 in contrast to 67 (23.0 %) in Syn4 (chi-squared test, *P* = 0.01) (Additional file 11). The average percentage of markers inherited from B73 in IBM Syn10 was higher than that in IBM Syn4 (53.51 % *vs.* 51.44 %, *U*-test, *P* = 9.74 × 10^−5^, Additional file 12).

The expected expansion factors were 6.5 and 4 for the IBM Syn10 and Syn4 populations, respectively. The maps were adjusted to F_2_-based maps. The length of the adjusted map of IBM Syn4 was 1,702.7 cM, which was consistent with previous reports (1,689 cM) [40]. The observed expansion factor of IBM Syn4 was 3.67 (6,242.7 cM/1,702.7 cM). The map of IBM Syn10 was adjusted directly by the expected expansion factor and the length of the adjusted map was 1,722.9 cM (Additional file 13).

### Genome-wide QTL analysis for plant height and flowering time

**Fig. 4:**
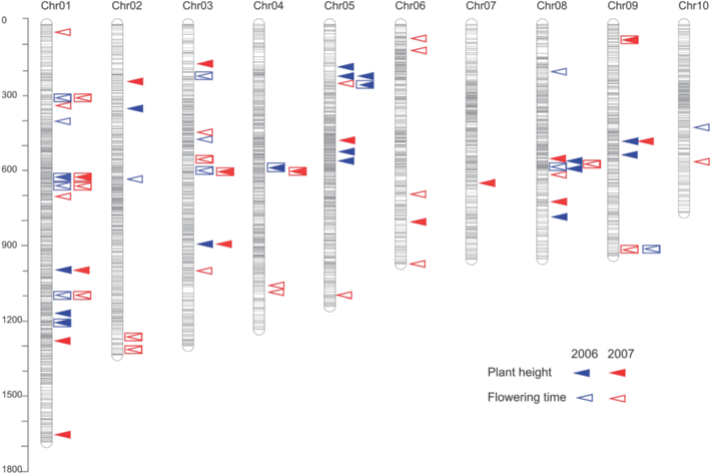
Genome-wide QTL mapping for flowering time and plant height traits in the IBM Syn10 population. The triangles on the right of the 10 chromosomes represent QTLs of plant height (solid) and flowering time (hollow) in the year 2006 (blue) and 2007 (red). The triangles surrounded by quadrilateral represent the QTLs detected in both IBM Syn10 and Syn4 populations

In total, we detected 135 QTLs for plant height and flowering time in the Syn4 and Syn10 populations. In the Syn4 population, 34 QTLs for flowering time were detected, including seven pairs of QTLs that overlapped on chromosomes 1, 4, 8, 9, and 10 among different years, whereas thirty-two QTLs for plant height were detected on chromosomes 1, 2, 3, 4, and 9. In the Syn10 population, 35 QTLs were detected for the flowering time, which included five pairs of QTLs that overlapped on chromosomes 1, 8, and 9 during different years; 34 QTLs were detected for plant height, which included six pairs of QTLs that overlapped on chromosomes 1, 3, 4, 8, and 9 (Fig. 4). By comparing the Syn4 and Syn10 populations, we found eight overlapping genome regions on chromosomes 1, 2, 3, 8, 9, and 10 for the flowering time QTL and five regions on chromosomes 1, 3, 4, and 9 for plant height QTL across various environments. The Syn4 population exhibited a 2.6- to 3.03-fold increase in the physical confidence interval compared to the Syn10 population when comparing the median values for those two traits (Table 1). A total of 28 QTLs in Syn10 (40.6 %) spanned a physical interval of less than 1 Mb, in contrast to eight QTLs (11.8 %) in Syn4. A total of 31 QTLs (44.9 %) spanned a physical interval from 1 Mb to 5 Mb in Syn10 and 30 QTLs in Syn4 (44.1 %). Ten QTLs (14.5 %) exceeded a physical interval size of 5 Mb in Syn10 and 28 QTLs in Syn4 (42.4 %) (Additional file 14).

**Table 1:** Summary of QTLs identified for flowering time and plant height traits for IBM syn4 and syn10 population

Treatment	QTL number	LOD thresholds			R^2^(100 %)^a^			Confidence interval (physical, Mb)^b^			Confidence interval (genetics, cM)^b^		
		Range	Average	Median	Range	Average	Median	Range	Average	Median	Range	Average	Median
Syn4-PH	32	2.51–14.37	5.36	4.53	2.82–14.15	5.46	5.21	0.09–13.31	5.77	4.19	4.4–57.3	17.41	15.15
Syn10-PH	34	2.59–10.31	4.67	4.15	3.3–14.5	6.08	5.19	0.3–10.15	2.21	1.38	8.75–22.6	14.51	13.7
Syn4-FT	34	2.49–8.61	4.47	4.17	2.7–11.65	5.4	5.13	0.3–9.25	5.64	4.81	6.1–46.9	16.72	14.25
Syn10-FT	35	2.57–17.89	5.14	4.41	2.82–24.06	6.29	5.12	0.2–52.7	4.96	1.85	6.65–43.35	14.31	12.4

^a^The percentage of the phenotype variation explained by QTL

^b^The phyiscal and genetics length of 2-LOD confidence interval of QTL

### High-quality SNP recalling and integrated map construction

**Fig. 5:**
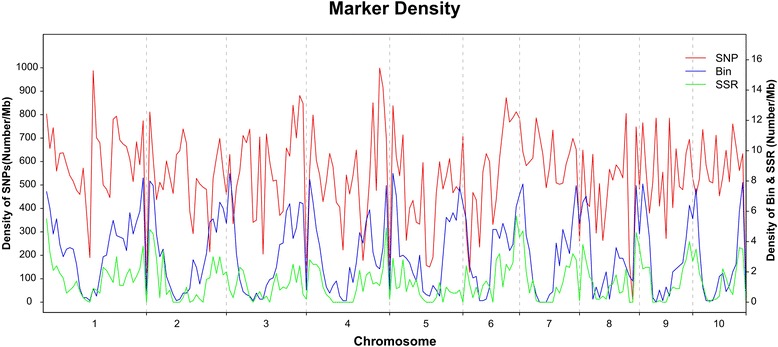
Marker density of integrated map consisting of 2,916 traditional (SSR/IDP) markers, 6618 bin markers, and 1,151,856 high quality SNPs. X-axis: physical position on 10 chromosomes. Left Y-axis: density of SNP markers (red line). Right Y-axis: density of bin markers (blue line), and SSR marker (green line). The marker density on the genome was calculated by sliding non-overlapping windows using window size of 10 Mb

A total of 1,151,856 high-quality SNPs were generated from the parental SNP set. In total, 76.5 % of the SNPs (1,505,749 SNPs) were verified at least once from the 280 lines in the Syn10 population, and 23.5 % of the SNPs were excluded because no SNPs were successfully validated from the Syn10 population. In our database, 18.77 % (216,254 out of 1,151,856 SNPs) were verified 1–4 times in the Syn10 population. Additionally, 81.23 % (935,602 of the verified high-quality SNPs) were verified more than five times in the Syn10 population. Finally, a total of 2,961 SSR, RFLP, and IDP markers with precise physical coordinates as well as extra 6,618 bin markers and 1,151,856 SNPs were collected to create a high-quality and density-integrated map (Fig. 5, Additional file 15).

## Discussion

### High-quality SNPs as a community resource for maize breeding in the iPlant platform

The iPlant Collaborative (iPlant) provides with open data access, pipeline process, and computing power. All of the data presented in this study can be found and re-analyzed via the iPlant Discovery Environment (DE). All of the SNPs between parents were verified in the IBM Syn10 population. Among the 974 marker genotype combinations that were verified by Sequenom MassARRAY, 919 (94.4 %) showed genotypes consistent with the data from re-sequencing and Sequenom-based genotyping (Additional file 16). We compared our SNP data from Mo17 with the data that were available in the MaizeGDB database. From our data set of 1,151,856 high-quality SNPs, 893,224 (77.6 %) SNPs had records in MaizeGDB, whereas 258,632 (22.4 %) SNPs were found to be novel. The high content of repeats in the maize genome introduces a high error probability in the process of SNP calling, owing to possible incorrect alignments [15]. In this study, 889,085 (99.54 %) of the overlapping SNPs between MaizeGDB Mo17 SNPs and our dataset had consistent genotypes. In addition, our Mo17 data were compared with Maize HapMap2 data [41], and 1,112,192 (96.56 %) SNPs have been reported in HapMap2. Among the reported SNPs, 916,746 (82.43 %) SNPs of Mo17 showed consistent genotypes in our study, and 177, 836 (15.99 %) were missing in Mo17 of HapMap2 (shown as “N”); only 17,610 (1.53 %) showed inconsistent genotypes (Additional file 17). In summary, our Mo17 SNP set is an important complement for data that were previously reported, and the new reliable SNP dataset has been combined with both bin markers and traditional markers to construct an integrated map, which will be valuable for further applications such as molecular breeding.

### Map expansion and inadvertent selection

Map expansion is a common phenomenon in genetic research [42, 43]. The observed recombination fraction of linked loci and the increasing map length with each generation are caused by the accumulation of crossovers during meiosis. An expanded map that is robust for QTL mapping reflects the actual recombination fractions. Adjusted F_2_-based maps are convenient for the comparison of different populations. Although IBM Syn4 and Syn10 have different lengths of their expanded maps, similar lengths of the adjusted maps were expected because they were derived from the same parents. Winkler *et al.* have deduced an equation for RIL and IRIL, which was appropriate for IBM Syn4 [44]. Because IBM Syn10 was constructed by producing doubled haploids as a final step, the theory equation was not suitable for this study. In a previous simulation study, the length of the expanded map tended to be underestimated when the marker density was not saturated. This bias disproportionally decreased with increasing marker density [45]. Consistently with previous simulations, the observed expansion factor of IBM Syn4 was lower than the expected expansion factor (3.67 *vs.* 4) under the limited density of genetic markers, and a similar underestimation has been reported in mice [43]. For IBM Syn10, this bias can be ignored given the higher marker density. Thus, the genetic coordinates of the markers in the IBM Syn10 map were adjusted by the expected map expansion factor. This treatment was justified by the results of our adjustment, in which comparable lengths of adjusted maps, 1,702.7 cM *vs.* 1,722.9 cM were obtained for IBM Syn4 and Syn10, respectively.

Segregation distortion has been widely reported in mapping populations, which distorts the frequency of the genotypes from the expected Mendelian ratio [46, 47]. Segregation distortion was observed in the present analysis and was associated with the number of markers in both the Syn4 and Syn10 populations. The distorted markers showed the same preferential segregation in most parts of the genome (Additional file 18). Furthermore, the Syn10 markers were subjected to more severe segregation distortion, and the average percentage of markers inherited from B73 in Syn10 was higher than that in Syn4. We inferred that the higher segregation distortion of Syn10 results from a greater number of inter-mating generations and meiotic events driving in the process of double-haploid formation [48]. Although there was more severe segregation in Syn10, there was not an obvious difference in the results of the QTL mapping between the two populations. Similar numbers of QTL were detected, and there was no significant difference in the average QTL effect between the two populations (*T*-test, P > 0.05). This finding was consistent with the previous conclusion that the effect of distortion could be ignored in large mapping populations [49, 50].

**Fig. 6:**
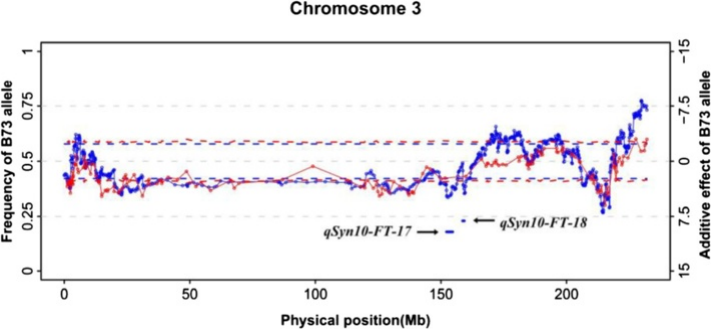
Overlap of orientation, physical coordinate of segregation distortion regions, and additive effect of two flowering time QTLs on chromosome 3. X axis: physical coordinate on the B73 chromosomes. Left Y axis: B73 allele frequency in IBM populations. Right Y axis: The additive effect of flowering time of QTL inherited from B73. Blue hollow circles and red hollow circles represent markers of IBM Syn10 and Syn4, respectively, B73 allele frequency of each marker was obtained by dividing the number of progeny by genotype of B73 allele using total number of progeny with non-missing genotype. Adjacent circles from the same population were connected by solid line to show variation tendency of B73 allele frequency among the genome. Blue and red dashed lines represent 99 % confidence interval for the “no distortion” hypothesis in the Syn10 and Syn4 populations, respectively. The information of two flowering time QTL such as physical coordinate, length, additive effect were indicated by two blue thick lines

In many plant species, the most commonly reported genetic factors associated with a distorted segregation ratio are the genes associated with gametophytes or sterility [51, 52]. In maize, inadvertent selection is potentially preferred to healthy and early flowering plants [53]; as a result, the flowering time was also a potential factor for segregation distortion [54]. A total of 278 and 122 markers were located in flowering time QTLs, and 153 and 34 markers were located in segregation distortion regions (SDRs) in IBM Syn10 and Syn4, respectively. Enrichment of distorted markers in regions of flowering time QTL were not significant compared to the whole genome in both populations (chi-squared test, *P* > 0.05). By further investigating 13 QTLs under most severe segregation distortion (*P* < 1.0 × 10^−5^ in the distortion test), the correlated relation between the orientation of the QTL additive effect was observed to be correlated with that of the segregation distortion. In both populations, the genotype of markers in these QTLs preferred to exist in the parent with a negative additive effect in the corresponding QTL (Fig. 6, Additional file 18). The only one exception was the QTL (*qSyn10-FT-35)* on chromosome 10 that showed a preference toward the B73 genotype, which had a positive additive effect in the QTL.

Markers in flowering time QTL did not show more universal segregation distortion compared to the whole genome in our study. We inferred that the additive effect of most flowering time QTLs varied widely under different environments; as a result, the effect of inadvertent selection did not accumulate over generations for most flowering QTLs. Only the QTLs with stable effects were under continuous inadvertent selection, and the genotype of these QTLs preferred to the parent with a negative additive effect, which could shorten the flowering time. This phenomenon was especially obvious on chromosome 3. There were two SDRs in the region of 140 ~ 150 Mb on chromosome 3 and both SDRs preferred the genotype of Mo17. Two QTLs, *qSyn10-FT-17* and *qSyn10-FT-18,* dramatically overlapped with the peak of these two SDRs (Fig. 6); meanwhile, Mo17 had a negative additive effect on the two QTL (positive for B73). It is plausible that such SDRs were driven by inadvertent selection for flowering time. Only the segregation distortion of the exceptional QTL *qSyn10-FT-35* appeared to be driven by some other factors, such as gametophyte genes.

### Superiority in the bin map of IBM Syn10

In rice, previous studies [30, 34] demonstrated that the bin map strategy offered complete genome coverage and was cost efficient and time saving compared to traditional molecular marker methods for detecting QTLs. In these rice studies, several high-density genetic maps (with an average physical interval of ~0.2 Mb between bin markers) were constructed with an average of ~0.05× genome coverage. Compared with previous reports, the bin map constructed in our study increased the marker density: the average ~0.31× genome coverage was expected to be sufficient for detecting most recombination events. In spite of a nearly six-fold increase in genome coverage compared to a previous study in rice (0.31/0.05), some regions in Syn10 still exhibited longer physical lengths between bin markers, especially in the regions near the centromeres of chromosomes 1, 4, 6, 7, 9, 10, with ten larger bins that ranged from 11.2 Mb to 18.8 Mb. This finding can be explained by the lack of recombination around centromeres [40, 55].

The length of the observed map of the IBM Syn10 population was longer than the previously reported lengths of the IBM Syn4 and F_2_ populations [29] (Additional file 19). As a result of map expansion, higher QTL mapping resolution was expected in the Syn10 population. This finding was verified by a comparison of the QTL interval lengths of the Syn4 and Syn10 populations. The median interval length of the plant height and flowering time QTL of Syn10 was 0.33- and 0.38-fold lower than in Syn4, respectively (Table 1). I.e. the QTL mapping resolution of Syn10 showed a three-fold increase compared to Syn4, with only a 1.79-fold increase in the length of the genetic map. Given that the effective population size of Syn10 for QTL mapping was smaller than the Syn4 population (194 *vs.* 244), the extra increase in the mapping resolution was due not only to the higher recombination fraction in Syn10 but also the higher marker density.

### Accuracy of QTL fine mapping in the Syn10 population and identification of candidate genes

**Fig. 7:**
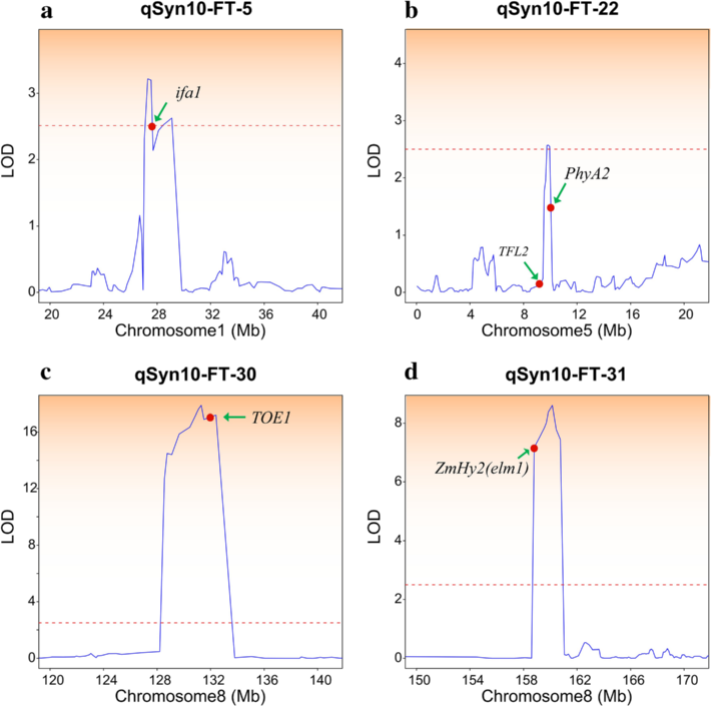
Precise location of 4 QTL harboring 5 cloned genes. The head of each sub-plot is the code name of QTL. Curves in plot indicate the physical coordinate (X-axis) and LOD (Y-axis) values of detected QTL. The red dash line indicates the LOD threshold (LOD=2.5). Arrowheads represent the relatively physical position of a cloned gene. qSyn10-FT-5 (**a**), qSyn10-FT-22 (**b**), qSyn10-FT-30 (**c**) and qSyn10-FT-31 (**d**) indicate the QTLs detected in IBM Syn10 for flowering time

Based on the positional cloning of QTL, we were interested in determining the genetic resolution in IBM Syn10. To verify the accuracy of the QTL detection, we used 177 published genes, including cloned and functionally verified genes, candidate genes from genome-wide association studies (GWAS), and genes that are homologous to known genes from other species (Additional file 20). Across all of the QTL across the two traits, we found a total of 18 cloned genes in the QTL regions, including 16 cloned genes for the flowering time and two for the plant height. Two known genes, *TFL2* and *PhyA2* (Fig. 7b), both of which encode the heterochromatin protein 1 homolog and repress flowering, were detected in a 0.7-Mb region on chromosome 5. The *ZmHy2 (elm1)* (Fig. 7d) gene, which encodes a phytochromobilin synthase, was detected and constrained to a 1.95-Mb region on chromosome 8. Among QTLs with underlying cloned genes, most of the genes were constrained to 0.2 Mb to 7.2 Mb. For seven genes, the physical coordinates of the genes did not match well with the QTL position (Additional file 21).

**Fig. 8:**
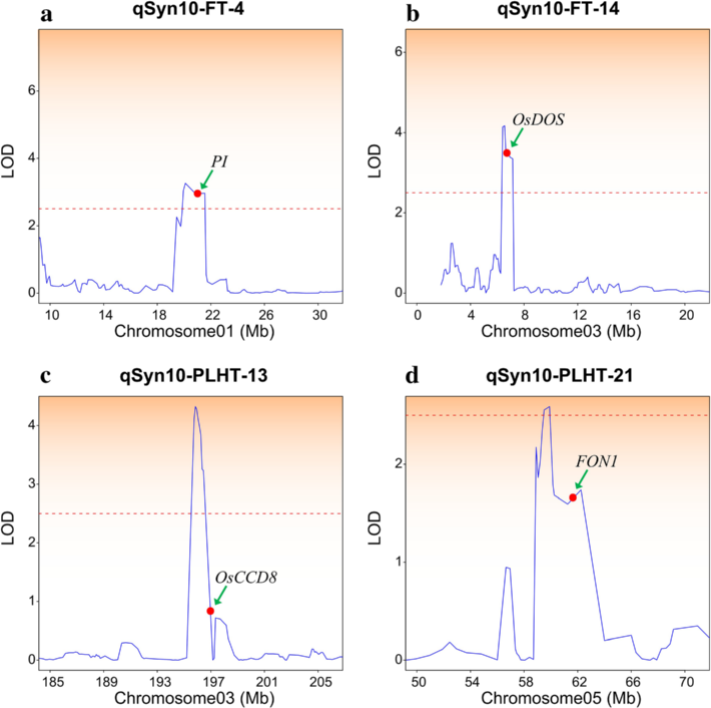
Precise location of 4 QTL harboring 4 candidate genes. The head of each sub-plot is the code name of QTL. Curves in plot indicate the physical coordinate (X-axis) and LOD (Y-axis) values of detected QTL. The red dash line indicates the LOD threshold (LOD=2.5). Arrowheads represent the relatively physical position of a candidate gene. qSyn10-FT-4, 14 indicated the QTLs detected in IBM Syn10 population for flowering time (**a** and **b**), qSyn10-PLHT-13, 21 indicated the QTLs detected in IBM Syn10 population for plant height (**c** and **d**).

With the integration of linkage mapping and comparative mapping, 25 candidate genes were mapped based on previous studies, including rice genes that affect the flowering time and plant height (Additional file 21). On chromosome 1, ranging from 14,850,871 bp to 20,995,107 bp, three candidate genes overlap with QTLs (*qSyn10-FT-2* and *qSyn10-FT-4*). The candidate gene *DOF* (GRMZM2G162749) encodes a zinc-finger protein, *PI* (GRMZM2G111191) (Fig. 8b), which is related to the *Pistillata* gene, a MADS-box gene, and the candidate gene *GWAS-DAT-1* (GRMZM2G000741), which is in the mitochondrial carrier family. In summary, a densely mapped IBM Syn10 population will help to identify and limit the number of positional candidate genes in QTL studies and identify promising candidates based on the sequence homology to characterized genes as a result of the availability of the B73 genome sequence.

## Methods

### Plant material and DNA isolation

Genotype data for a set of 244 IBM Syn4 RILs are available at (Additional file 13) [25, 29]. The IBM Syn10 DH lines were developed from maize inbred lines B73 and Mo17 with ten generations of random mating, which were inter-mated after the F_2_ (Additional file 1). In this study, 280 of the IBM Syn10 DH lines (Hussain et al. [25]) were used for genotyping, whereas 194 of the 280 lines were only used to collect phenotype data. Both IBM Syn10 and Syn4 populations were planted in separate but adjacent fields at the Agronomy Agricultural Engineering Research Center, Ames, Iowa, in 2006 and 2007. Growing degree days (GDD) were calculated in °C day from planting until the date when at least 50 % of the tassels in the plots were shedding pollen. The plant height (PH) was measured from the soil surface to the flag leaf collar on five representative plants within each plot. In 2011, 280 of the IBM Syn10 DH lines and Mo17 were planted in the Agronomy Greenhouse of Iowa State University for genomic DNA extraction using the CTAB method as previously described [56].

### Sequencing and short-read alignments

At least 5 μg of genomic DNA from each sample was randomly fragmented by sonication and ~500 bp DNA fragments were purified and size-selected to construct library. Adapter ligation and DNA cluster preparation were performed, and sequencing was conducted by using an Illumina Hiseq2000 instrument according to the manufacturer’s instructions (Illumina, San Diego, CA). Indexed DNA samples of eight IBM lines were combined and sequenced per lane. The short reads were aligned to the maize reference genome (B73 RefGen_v2) [16] using *SOAPaligner*, version 2.21 (*SOAP2* parameters: −m 283 -x 514 -s 40 -l 32 -v 5) [57–60]. Based on the mapping results, the alignment reads and unique hit reads in each sample were classified by *SOAP2*.

### SNP detection and annotation of Mo17

The same procedure for detecting high-quality SNPs between parents of Mo17 and B73 was used as previously described [32, 61]. In summary, we first calculated the likelihood of the Mo17 inbred line’s genotype using *SOAPsnp* and obtained the consensus sequence (pseudo-genome of Mo17). Second, polymorphic loci compared to the B73 reference sequence were extracted using the consensus sequence. Finally, SNP determination was performed only for sites with sufficient quality (effective sites).

Candidate SNPs having sufficient quality based on the following criteria were kept: 5 ≤ depth ≤ 100, average mappable sites < 1.5, and a sufficient quality score (average quality for the novel allele ≥ 20). To exclude SNP calling errors caused by incorrect mapping or indels, adjacent SNPs that were separated by < 5 bp were not called. These SNPs were used to construct the whole-genome SNP and bin map [60, 62]. The localization of SNPs in annotated regions was based on B73_RefGen_v2 in the Phytozome 7.0 database (ftp.jgi-psf.org/pub/compgen/phytozome/v7.0/Zmays/annotation/Zmays_166_gene_exons.gff.gz) [32].

### Short-indel detection of Mo17

Small insertions and deletions (indels 1–5 bp) were detected by mapping the reads to B73 (allowing gaps) using *SOAPindel*, version 2.0 (http://soap.genomics.org.cn/soapindel.html). For short-indel detection, the paired-end reads that contained alignment gaps at only one end were allowed. Short indels were identified in three subsequent steps: (1) mapping the paired-end reads to the B73 reference sequence by fitting up to a 5-bp gap, (2) merging the redundant pairs prior to searching for indels, and (3) extracting of gaps that were supported by at least three non-redundant paired-end reads and had a quality score (provided by *SOAPindel*) higher than 19 [32, 63].

### Genotyping and bin map construction of IBM Syn10

For each DH line, the 90-bp reads (83-mers, excluding the index) were sorted according to 5′ indexes. In average, ~640 Mb sequences were generated for each DH line, which was equivalent to 0.31× coverage of the B73 genome. The custom-made Mo17 pseudomolecules were generated by replacing B73 pseudomolecule sequences with those of Mo17 at SNP sites. The reads from DH lines were aligned against the B73 and Mo17 pseudomolecules using *SOAPaligner,* respectively. The reads that mapped uniquely to either parent but with at most a 1-bp mismatch were called as high-quality SNPs. The reads that matched multiple locations or contained low-quality sequences (quality value ≤ 5) were discarded. Finally, a subset of SNPs, which excluded the low-quality SNPs, was aligned to the chromosomes according to their physical location [59]. Two custom Perl scripts were written to accurately detect the genotype for each genomic region and to identify recombination breakpoints.

Bin maps were then constructed for the 280 DH lines. Consecutive SNPs were examined with a sliding window approach (15 SNPs in size) and used to calculate the ratio between the numbers of SNPs from the two parents in each window. As the window slides along the chromosome, recombination breakpoints can be determined. The window size was dynamic depending on the SNP density within a given DH line [30, 64]. The map of each individual line was aligned, and genotypes were determined for each 100-kb interval. Adjacent 100-kb intervals with the same genotype in each line were merged into a bin using two custom Perl scripts.

A high-density genetic map was constructed for IBM Syn10 across all 6,618 recombination bins. The genotype of each recombination bin was represented by a bin marker, which was defined as a set of genetically linked SNPs of the same genotype that were collected into a single bin group with a physical position located in the middle of each recombination bin, which was used as a virtual bin marker to genotype the population.

Two different software packages were used for genetic map construction: *JoinMap 4.1* [65] and *MSTMap* [66]. *JoinMap 4.1* was used for marker grouping using two-point maximum likelihood distance analysis. Ten linkage groups, along with one additional very small linkage group, were determined by applying an independent LOD (logarithm [base 10] of odds) value of 9. Initial map orders were assembled using *MSTMap* and the *Kosambi* map function using a maximum-likelihood algorithm because of the high computational efficiency of *MSTMap* [66]. The marker order of each linkage group was further optimized by *JoinMap 4.1* with the maximum-likelihood algorithm. Multipoint recombination frequencies were estimated by Gibbs sampling. Four Monte Carlo EM cycles with chain lengths of 2,500 were run after a burn-in period of 5,000 steps. The marker order was established by simulated annealing using a maximum-likelihood approach. Simulation began with an initial acceptance probability of 0.350 and a cooling control parameter of 0.001. The chain length of the iterations was 10,000. The chain with a constant acceptance probability was maintained, and the next chain was allowed to run with a smaller acceptable probability. The iterations were stopped after 5,000 chains without improvement. Recombination fractions of all pairs of markers were estimated and visualized in Additional file 17 to evaluate the quality of the genetic map.

### Segregation distortion and adjustment for map expansion

Segregation distortion was tested for 6,618 bin markers of the Syn10 population and 1,339 SSR markers of Syn4 using a chi-squared test. Among the progeny lines in both populations, a 1:1 segregation was expected for all of the polymorphic markers. We expected that, on average, across all of the markers and lines, both parents would contribute 50 % to their genomes. In the mapping population, the segregation of linked markers was not independent, and the proportion of significant markers was very large in our study (i.e. a false-positive rate that was much higher than expected). Thus, the effect of the false positives was expected to be limited, and we did not correct for multiple comparisons.

The genetic maps of both IBM Syn10 and Syn4 were adjusted to an F_2_-based map (only one generation of meiosis and crossover). The expected expansion factors were determined by the equation $\alpha =\frac{j}{2}+\frac{\left({2}^j-1\right)}{2^i},.$ where *j* is the number of intermating generations, including the two generations for creating the F_2_, and *i* is the number of inbred generations after intermating [45]. For IBMSyn4, j = 6 and i = ∞. For IBM Syn10, j = 12 and i = 1, because only one generation proceeded in the process of double-haploid development. The map of IBM Syn4 was adjusted according to the adjustment equation for intermated recombination inbred lines (IRIL) [44]. The map of IBM Syn10 was adjusted directly by using the expected expansion factor. Each genetic interval between markers was divided by the expected expansion factor to generate an adjusted map.

### QTL mapping and analysis

A total of 194 IBM Syn10 DH lines were used for QTL analyses due to a lack of phenotypic information in the remaining 86 lines. The genetic distances of the bin-markers were re-calculated among those 194 DH lines for the purpose of QTL mapping. Subsequently, QTL analysis was performed according to the observed expanded map by using *QTL Cartographer Unix version 1.17 f* [67]. Composite interval mapping for population type “RI0” was used (CIM; Zmap model 6). The ten most significant cofactors were identified by using forward and backward regression with a window size of 5 cM and a step size of 0.5 cM. QTLs with LOD values that were larger than 2.5 were called, for which the QTL location was determined, including 2-LOD drop support intervals. The same parameters, but for population-type “RIL,” were applied to QTL analysis of the IBM Syn4 population, which was genotyped with 1,339 markers (available at Additional file 13). The integrated version of iPlant CIM were parallelized for simultaneously analyzing multiple traits and chromosomes.

### Comparison of the QTL locations

The QTL locations of the two populations were compared according to the physical position of the flanking markers of each 2-LOD drop support interval. Only 435 markers (informative markers) had physical position information among the 1,339 markers used for analysis in the Syn4 population. The physical position of the remaining 904 non-informative markers was imputed. First, the maize genome was separated into regions on the basis of informative markers, and the average recombination rate (Mb/cM) of each region was calculated as the quotient of the physical distance divided by the genetic distance of two adjacent informative markers. The physical position of non-informative markers within each region was assigned according to its genetic distance relative to the informative markers and the average recombination rate of the region.

### Integrated map construction

The SSR and IDP markers for the integrated map were collected from the MaizeGDB website (http://www.maizegdb.org/cgi-bin/coordinates/map_regions.cgi), which was previously published as an ISU Integrated IBM 2009 map. MaizeGDB (http://www.maizegdb.org/) was used to recalculate the map distance via BLAST on B73 RefGen_v2 by sequence similarity. Hence, we collected only the markers with a unique position in the B73 reference genome. Bin marker data were calculated based on the physical position as described above. SNP markers were verified and filtered by identity by descent (IBD) in Syn10 lines before they were integrated into an integrated map: (1) For each Syn10 line, the expected genotype was assigned to SNPs in each recombination bin according to the genotype of the bin marker because SNPs and the corresponding bin marker were IBD. (2) For each Syn10 line, the observed genotype of the SNPs were obtained from sequencing data; only SNPs with consistently expected and observed genotypes were maintained, and inconsistent SNPs were discarded. (3) For a SNP marker between parents, if one progeny line showed a consistent genotype at this locus, then this locus was judged to be verified once. Only the SNP loci with at least one verification were retained as high-quality parent SNPs. Finally, we used the physical coordinates of SSR, IDP, bin, and high-quality parental SNP markers to create a high-quality and density-integrated map by using custom Perl and Shell scripts.

### High-quality parent SNP verification

To verify the accuracy of SNP genotypes among the parents and progeny in Syn10, we randomly selected 100 SNPs from the whole genome. The Sequenom primer design software (MassARRAY Assay Design 3.0) was employed for SNP primer design. The two parents and ten random progenies of Syn10 were selected for genotyping. Experiments were conducted according to the Sequenom iPLEX Assay application protocol [68]. Genotyping data were acquired using the Sequenom MassARRAY and processed using Sequenom Typer3.4 software [68]. Among the 100 SNPs, 91 SNPs were successfully designed and genotyped. Among the 1,092 (91 SNPs × 12 samples) marker genotype combinations, 44 generated missing data, and 74 resulted in heterozygous genotypes (Additional file 18). The parents and DH progenies were expected to be homozygous, especially the progenies. Therefore, the heterozygous genotypes were considered to be genotyping errors. The missing and heterozygous genotypes from this Sequenom genotyping were discarded, leaving 974 effective sites for verification. The high-quality SNPs of Mo17 were compared with those available in the MaizeGDB database and the maize Hapmap2 database.

### Data access

The re-sequencing data for Mo17 and the IBM Syn10 population have been deposited in the NCBI Sequence Read Archive (SRA) (http://www.ncbi.nlm.nih.gov/sra) under accession number SRP018832. The SNPs, bin markers, linkage maps, trait data, and parallelized CIM are accessible through the iPlant DE (http://data.maizecode.org/maize/qtl/syn10/).

## Conclusion

In briefly, our founding provides high value basis data for researchers not only for QTL mapping but also in chip building for molecular breeding. 1) We combine a million of high quality SNPs, 2916 traditional markers and 6618 bin markers together for QTL mapping and molecular breeding. All of the SNPs which come from Mo17 and B73 had been carefully checked and part of them had been verified by using the Sequenom MassARRAY and presenting a high Accuracy rates in the dataset; 2) Fine mapping Eighteen functional known genes and twenty-five candidate genes by bin map strategy, Map expansion and segregation distortion were also analyzed, and evidence for inadvertent selection of early flowering time in the process of mapping population development were observed; 3) All of the data were public resource and easily used via the iPlant platform (http://data.maizecode.org/maize/qtl/syn10/). Overall, our findings provide a fundamentally genetic data for QTL mapping in an updated IBM Syn10 population and provide a reliable and verified high quality SNP set between Mo17 and B73 as a basic data resources for maize committee around the world.

## Additional files

Additional file 1: Figure S1. Flowchart of the development of IBM Syn4 (left) and Syn10 (right) population in the present study. Two populations were generated through similar process, but showed differences in the number of generations of inter-mating and the strategies of generating homozygous progenies after inter-mating. (TIF 796 kb) Additional file 2: Table S1. Summary of Mo17 production and alignment results. (XLSX 9 kb) Additional file 3: Figure S2. Homologous SNP distribution along chromosomes of maize. The physical coordinate (X-axis) of the 2,200,187 homozygous SNPs are plotted along the 10 maize chromosomes (10 bars paralleled to Y-axis) with blue short vertical solid lines. (TIF 924 kb) Additional file 4: Table S2. SNP discovery between Mo17and B73. (XLSX 10 kb) Additional file 5: Table S3. SNPs annotation in CDS region. (XLSX 13382 kb) Additional file 6: Table S4. Indel discovery between Mo17 and B73. (XLSX 10006 kb) Additional file 7: Table S5. Summary of production and alignment for 280 IBM Syn10 lines. (XLSX 36 kb) Additional file 8: Table S6. Statistics for bin map data. (XLSX 214 kb) Additional file 9: Figure S3. Comparison of genetic length of linkage group (LG) and genetic coordinate of markers in LG between the two populations. Each pair of vertical bars present pair of LG of Syn10 (left) and Syn4 (right) derived from the same chromosome. The horizontal thin lines on bars indicate the genetic coordinate of bin/SSR markers in LG of two populations. The scale of LG in centiMorgan is given on the left of figure. (TIF 503 kb) Additional file 10: Figure S4. Correction of a potential assembly error in B73 reference. A fragment consisting of 4 alignment based bins (600kb) from chromosome 8 was re-assigned to linkage group 2 (chromosome 2) by following linkage mapping. (PDF 36 kb) Additional file 11:
**Statistics and comparison of Marker segregation distortion and genetic composition among IBM Syn4 and Syn10 populations.** (A) The comparison of the percentage of markers in segregation equilibrium and distortion .The markers were classified in to the groups of segregation equilibrium and distortion (chi-squared test, threshold: *P*=0.01), then the marker of segregation distortion were further divided into groups of “Bias to B73”and “ Bias to Mo17” according to observed genotype frequency compared with expected frequency; (B) The comparison of the percentage of line in different preference of genetic composition. The genetic composition of each lines were classified into the groups of composition equilibrium and distortion (chi-squared test, threshold: *P*=0.01), then lines of segregation distortion were further divided into groups of “Bias to B73”and “ Bias to Mo17” according to observed proportion of genetic composition compared with expected proportion. (TIF 235 kb) Additional file 12: Figure S6. Genetic composition of IBM Syn4 and Syn10 population. X axis: one vertical bar present one progeny in the corresponding population. Y axis: The percentage of markers of each progeny inherited from bi-parents. For each progeny, the genetic origin of each marker was identified according to the genotype of the marker in bi-parents and progeny. The length of blue and red parts of each vertical bar represent the percentage of markers inherited from B73 or Mo17, respectively. The genetic composition of IBM Syn10 was more biased toward B73 (the average percentage of markers inherited from B73 in IBM Syn10 was higher than in IBM Syn4, U-test, *P*=9.74 × 10^−5^). (PDF 47 kb) Additional file 13: Table S7. Genetic and physical coordinate for Syn4 and Syn10 population. (XLSX 13078 kb) Additional file 14: Table S8. Genome wide QTL identification between IBM Syn4 and Syn10 populations. (XLSX 118 kb) Additional file 15: Table S9. Integrate map with high quality SNPs, bin markers and traditional markers. (XLSX 28 kb) Additional file 16: Table S10. Verify the high quality SNPs between parents by using Sequenom MassARRAY. (XLSX 10 kb) Additional file 17: Table S11. Comparison of genotype of Mo17 (SNPs) in the current study and the maize hapmap2 database. (XLSX 19 kb) Additional file 18: Figure S7. Comparison of orientation of segregation distortion and additive effect of flowering time QTL on 10 chromosomes. X axis: physical coordinate on the B73 chromosomes. Left Y axis: B73 allele frequency in IBM populations. Right Y axis: The additive effect of flower time of QTL inherited from B73. Blue hollow circles and red hollow circles represent markers of IBM Syn10 and Syn4, respectively. B73 allele frequency of each marker was obtained by dividing the number of progeny with genotype of B73 allele using total number of progeny with non-missing genotype. Adjacent circles from the same population were connected by solid line to show variation tendency of B73 allele frequency among the genome. Blue dashed and red dashed lines represent the 99% confidence interval for the “no distortion” hypothesis in the Syn10 and Syn4 populations, respectively. The information of flowering time QTL such as physical coordinate, length, and additive effect was indicated by blue (Syn10) and red (Syn4) thick lines. (PDF 163 kb) Additional file 19: Figure S8. Comparison of the chromosome genetic length in F_2_, IBM Syn4 and Syn10 populations. (PDF 11 kb) Additional file 20: Table S12. The gene list for flowering time and plant height traits which collected from previous work (XLSX 28 kb) Additional file 21: Table S13. Fine mapping the cloned and candidate genes for flowering time and plant height in Syn4 and Syn10. (XLSX 19 kb)

## Abbreviations

IBM Syn10 DH population: intermated B73 × Mo17 synthetic 10 doubled haploid population
IBM Syn4 RIL population: intermated B73 × Mo17 synthetic 4 recombinant inbred lines population
QTLs: quantitative trait locus
DE: discovery environment
NGS: next-generation sequencing
CIM: composite interval mapping
SDRs: segregation distortion regions
GWAS: genome-wide association studies
GDD: growing degree days
PH: plant height
Indels: insertions or deletions
CDS: coding sequence
LG: linkage group
