# Supplementary figures and images for: An ultra-high-density map as a community resource for discerning the genetic basis of quantitative traits in maize
Source: BMC Genomics. 2015 Dec 21;16:1078. doi: 10.1186/s12864-015-2242-5 (PMC4687334; doi:10.1186/s12864-015-2242-5)

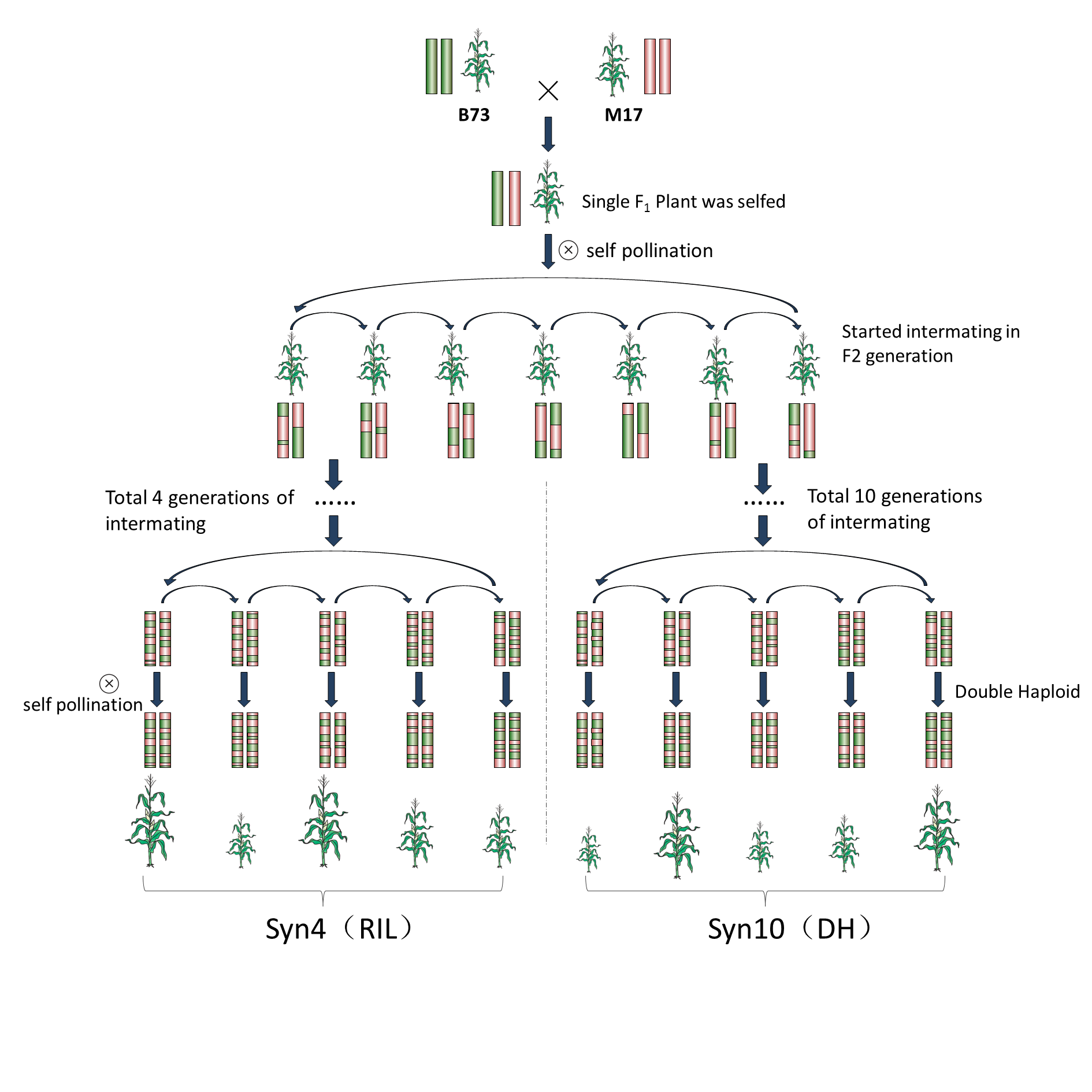

Supplement: Additional file 1: Figure S1. — Flowchart of the development of IBM Syn4 (left) and Syn10 (right) population in the present study. Two populations were generated through similar process, but showed differences in the number of generations of inter-mating and the strategies of generating homozygous progenies after inter-mating. (TIF 796 kb) [file 12864_2015_2242_MOESM1_ESM.tif]

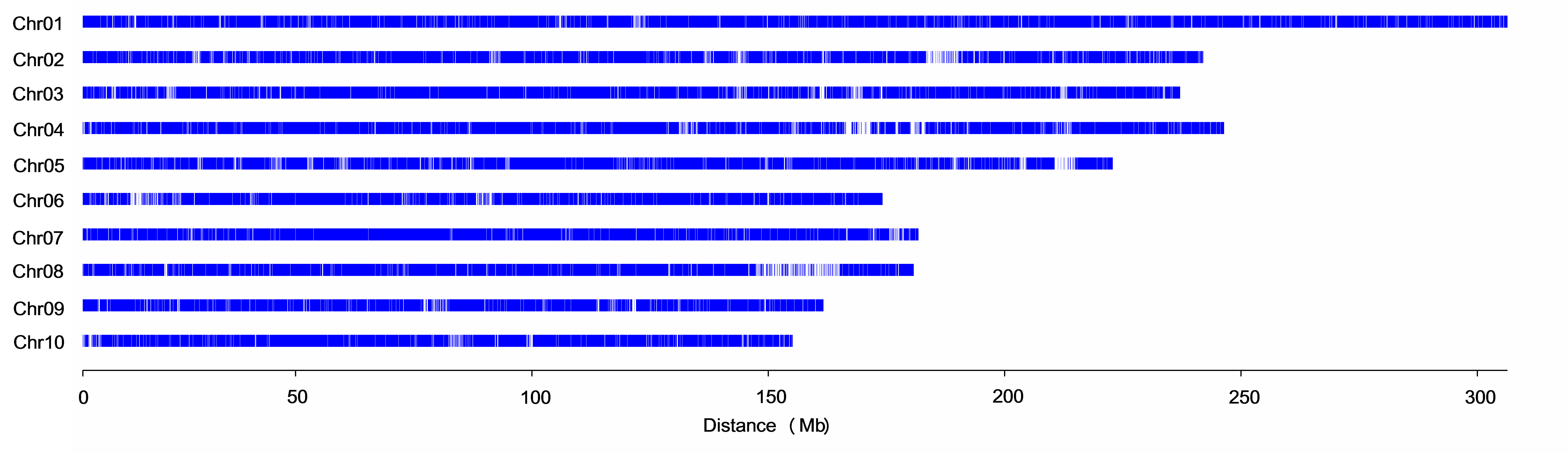

Supplement: Additional file 3: Figure S2. — Homologous SNP distribution along chromosomes of maize. The physical coordinate (X-axis) of the 2,200,187 homozygous SNPs are plotted along the 10 maize chromosomes (10 bars paralleled to Y-axis) with blue short vertical solid lines. (TIF 924 kb) [file 12864_2015_2242_MOESM3_ESM.tif]

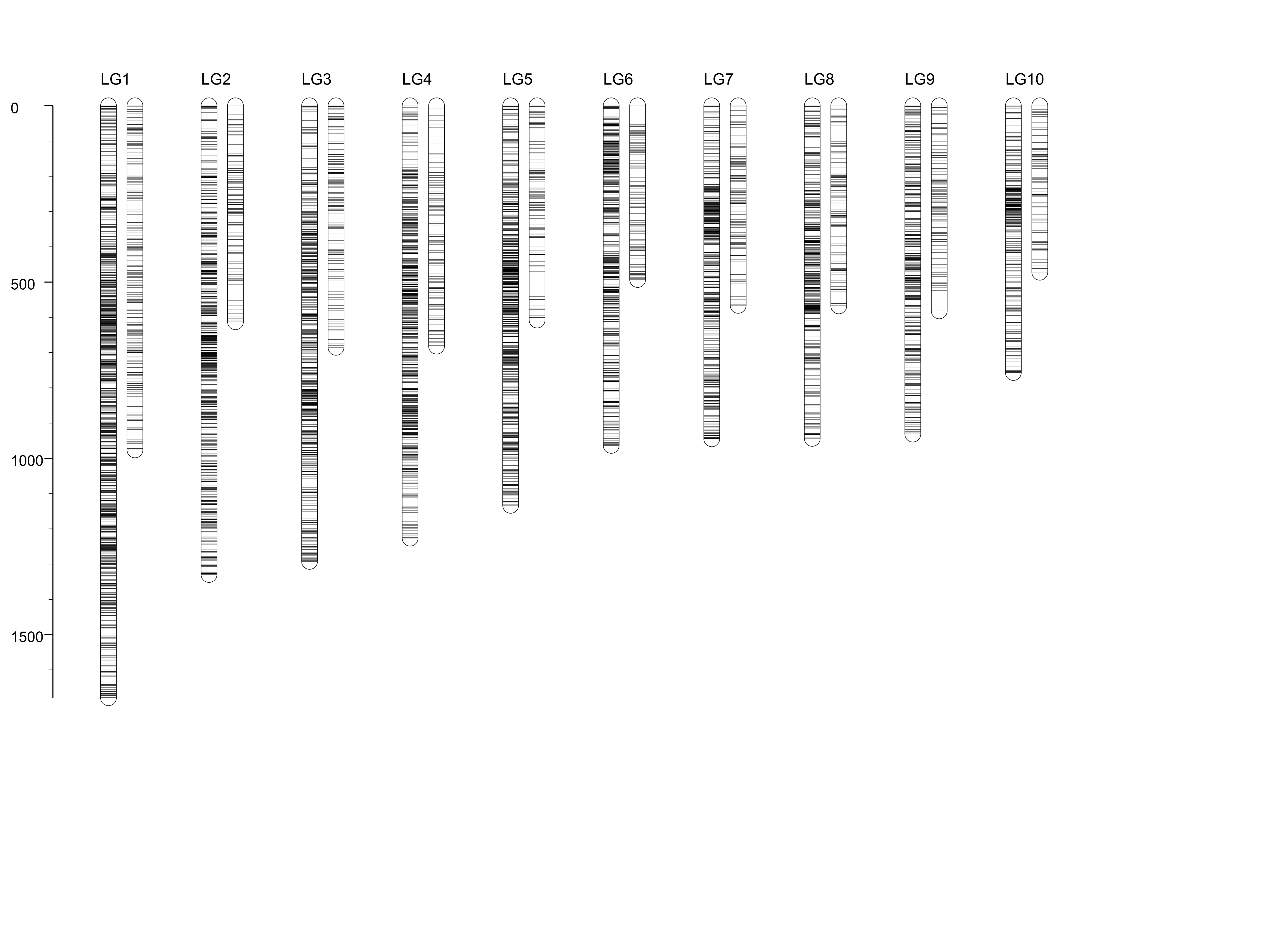

Supplement: Additional file 9: Figure S3. — Comparison of genetic length of linkage group (LG) and genetic coordinate of markers in LG between the two populations. Each pair of vertical bars present pair of LG of Syn10 (left) and Syn4 (right) derived from the same chromosome. The horizontal thin lines on bars indicate the genetic coordinate of bin/SSR markers in LG of two populations. The scale of LG in centiMorgan is given on the left of figure. (TIF 503 kb) [file 12864_2015_2242_MOESM9_ESM.tif]

Alignment based  
Bin map of  
chromosome 8

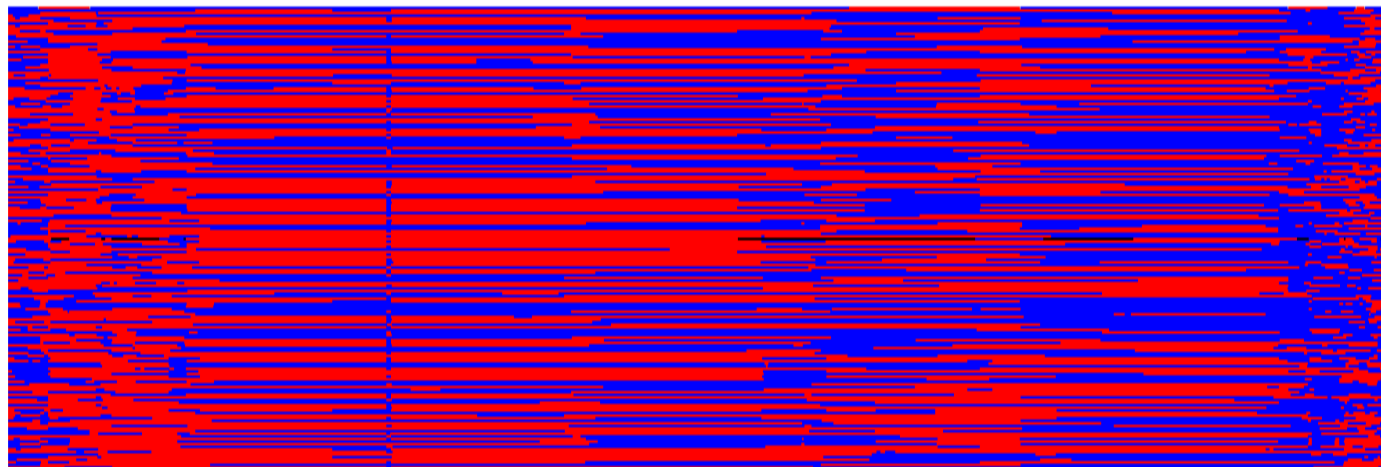

Re-assigned  
fragment

48.2

48.6 (Mb)

Bin marker ID

Chr08.482.5

Chr08.484.5

Chr08.487.5

Chr08.486.5

LG02

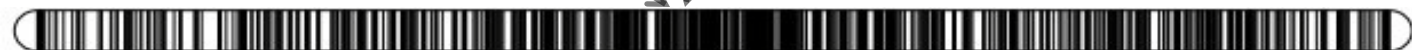

0

500

1000

cM

Supplement: Additional file 10: Figure S4. — Correction of a potential assembly error in B73 reference. A fragment consisting of 4 alignment based bins (600kb) from chromosome 8 was re-assigned to linkage group 2 (chromosome 2) by following linkage mapping. (PDF 36 kb) [file 12864_2015_2242_MOESM10_ESM.pdf]

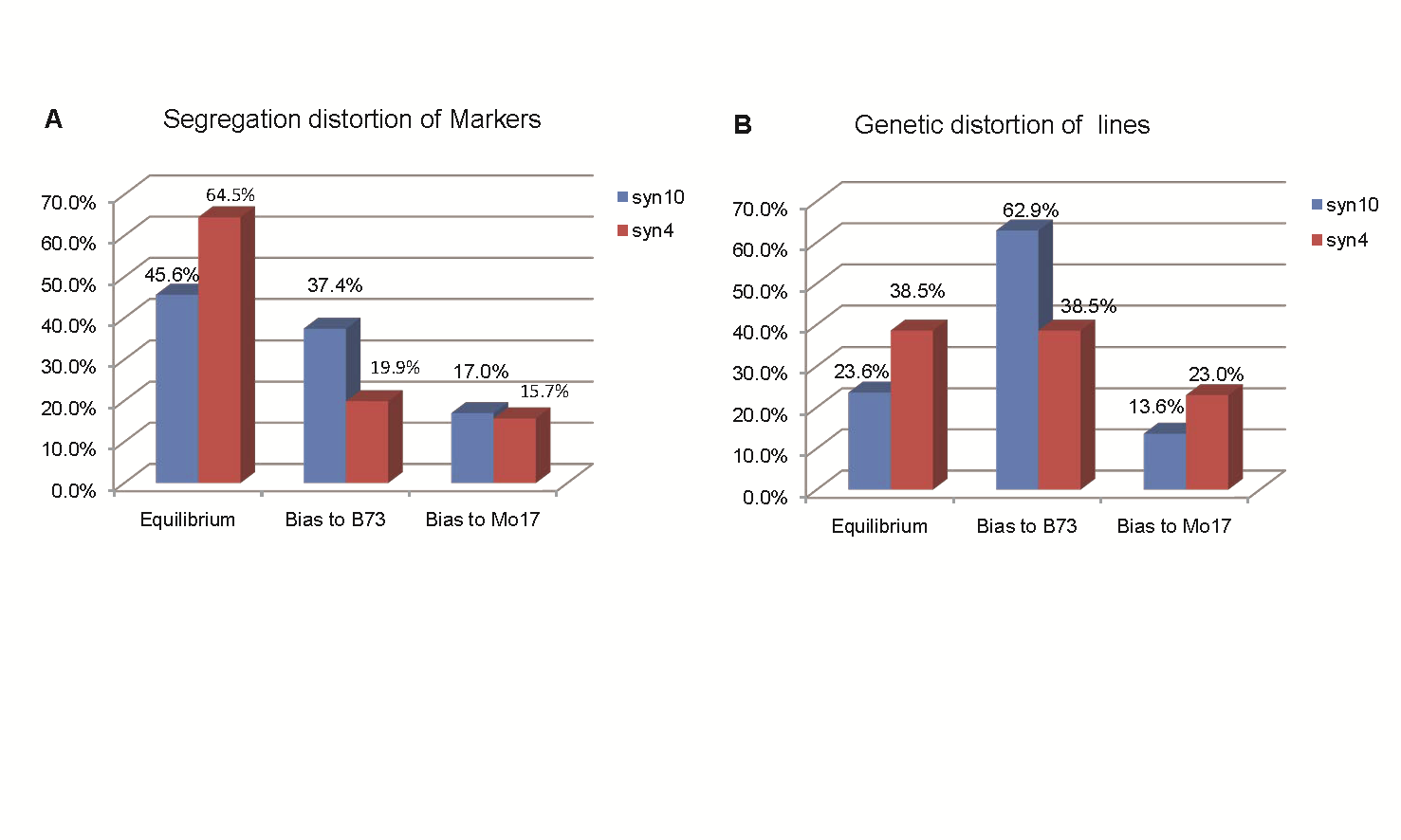

Supplement: Additional file 11: — Statistics and comparison of Marker segregation distortion and genetic composition among IBM Syn4 and Syn10 populations. (A) The comparison of the percentage of markers in segregation equilibrium and distortion .The markers were classified in to the groups of segregation equilibrium and distortion (chi-squared test, threshold: P=0.01), then the marker of segregation distortion were further divided into groups of “Bias to B73”and “ Bias to Mo17” according to observed genotype frequency compared with expected frequency; (B) The comparison of the percentage of line in different preference of genetic composition. The genetic composition of each lines were classified into the groups of composition equilibrium and distortion (chi-squared test, threshold: P=0.01), then lines of segregation distortion were further divided into groups of “Bias to B73”and “ Bias to Mo17” according to observed proportion of genetic composition compared with expected proportion. (TIF 235 kb) [file 12864_2015_2242_MOESM11_ESM.tif]

**Syn10**

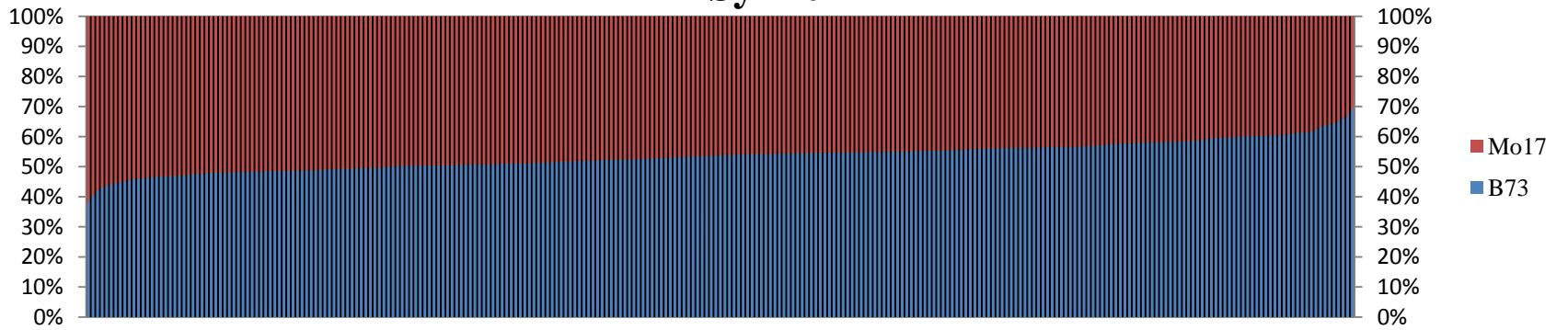

**Syn4**

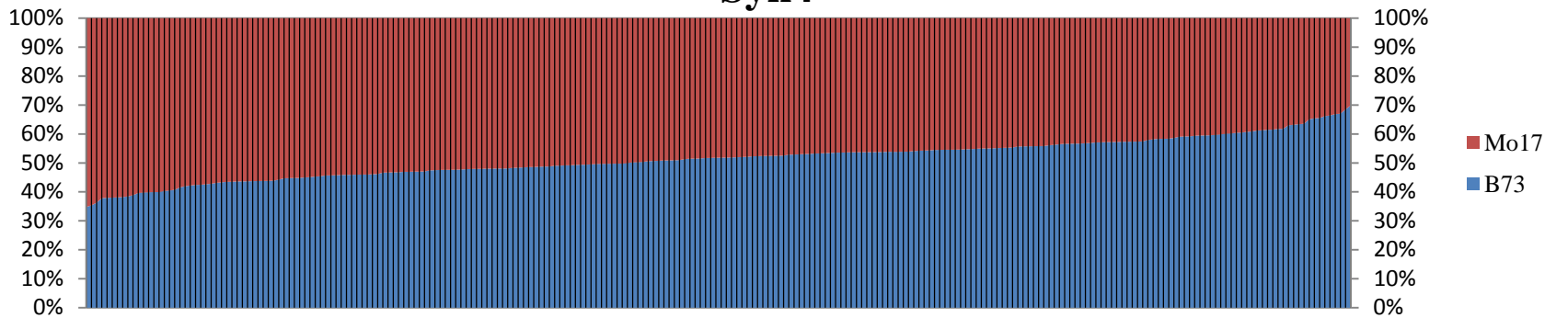

Supplement: Additional file 12: Figure S6. — Genetic composition of IBM Syn4 and Syn10 population. X axis: one vertical bar present one progeny in the corresponding population. Y axis: The percentage of markers of each progeny inherited from bi-parents. For each progeny, the genetic origin of each marker was identified according to the genotype of the marker in bi-parents and progeny. The length of blue and red parts of each vertical bar represent the percentage of markers inherited from B73 or Mo17, respectively. The genetic composition of IBM Syn10 was more biased toward B73 (the average percentage of markers inherited from B73 in IBM Syn10 was higher than in IBM Syn4, U-test, P=9.74 × 10^−5^). (PDF 47 kb) [file 12864_2015_2242_MOESM12_ESM.pdf]

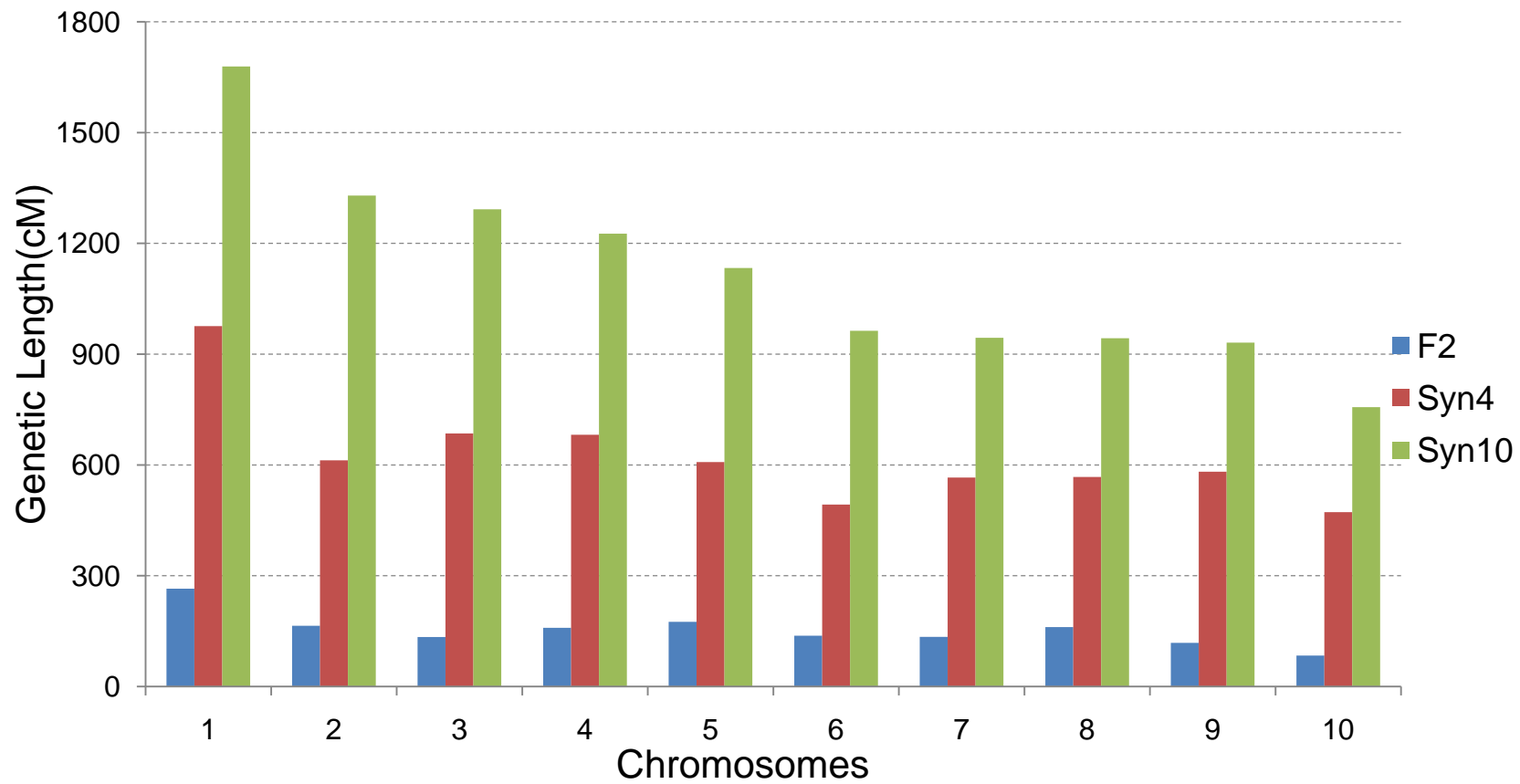

Supplement: Additional file 19: Figure S8. — Comparison of the chromosome genetic length in F2, IBM Syn4 and Syn10 populations. (PDF 11 kb) [file 12864_2015_2242_MOESM19_ESM.pdf]
