# Supplementary material for: An ultra-high-density map as a community resource for discerning the genetic basis of quantitative traits in maize
Source: BMC Genomics. 2015 Dec 21;16:1078. doi: 10.1186/s12864-015-2242-5 (PMC4687334; doi:10.1186/s12864-015-2242-5)
Supplement: Additional file 18: Figure S7. — Comparison of orientation of segregation distortion and additive effect of flowering time QTL on 10 chromosomes. X axis: physical coordinate on the B73 chromosomes. Left Y axis: B73 allele frequency in IBM populations. Right Y axis: The additive effect of flower time of QTL inherited from B73. Blue hollow circles and red hollow circles represent markers of IBM Syn10 and Syn4, respectively. B73 allele frequency of each marker was obtained by dividing the number of progeny with genotype of B73 allele using total number of progeny with non-missing genotype. Adjacent circles from the same population were connected by solid line to show variation tendency of B73 allele frequency among the genome. Blue dashed and red dashed lines represent the 99% confidence interval for the “no distortion” hypothesis in the Syn10 and Syn4 populations, respectively. The information of flowering time QTL such as physical coordinate, length, and additive effect was indicated by blue (Syn10) and red (Syn4) thick lines. (PDF 163 kb) [file 12864_2015_2242_MOESM18_ESM.pdf]

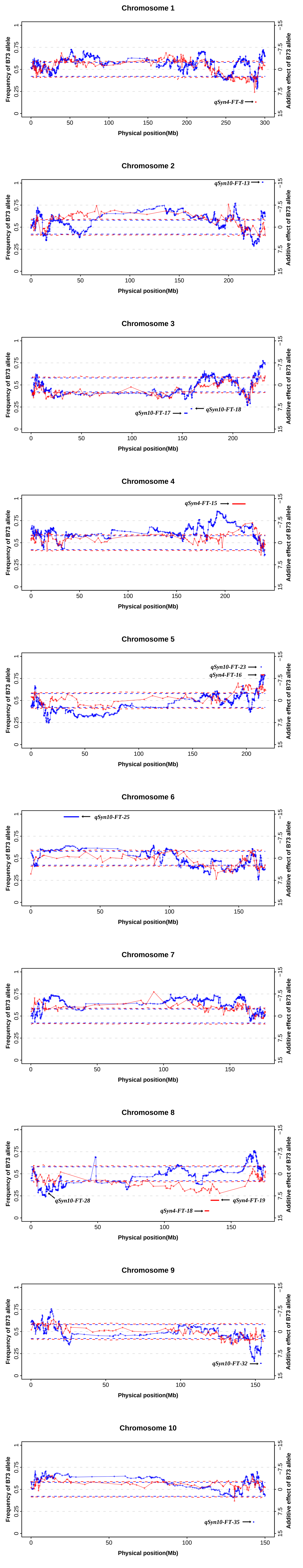
Chromosome 1
Frequency of B73 allele
Additive effect of B73 allele
Physical position(Mb)
qSyn4-FT-8
Chromosome 2
qSyn10-FT-13
Chromosome 3
qSyn10-FT-17
qSyn10-FT-18
Chromosome 4
qSyn4-FT-15
Chromosome 5
qSyn10-FT-23
qSyn4-FT-16
Chromosome 6
qSyn10-FT-25
Chromosome 7
Chromosome 8
qSyn10-FT-28
qSyn4-FT-19
qSyn4-FT-18
Chromosome 9
qSyn10-FT-32
Chromosome 10
qSyn10-FT-35
